# Effects of early life trauma are dependent on genetic predisposition: a rat study

**DOI:** 10.1186/1744-9081-7-11

**Published:** 2011-05-06

**Authors:** Toni-Lee Sterley, Fleur M Howells, Vivienne A Russell

**Affiliations:** 1 Department of Human Biology, Faculty of Health Sciences, University of Cape Town, Observatory, 7925, South Africa; 2 Department of Psychiatry, Faculty of Health Sciences, University of Cape Town, Observatory, 7925, South Africa

## Abstract

**Background:**

Trauma experienced early in life increases the risk of developing a number of psychological and/or behavioural disorders. It is unclear, however, how genetic predisposition to a behavioural disorder, such as attention-deficit/hyperactivity disorder (ADHD), modifies the long-term effects of early life trauma. There is substantial evidence from family and twin studies for susceptibility to ADHD being inherited, implying a strong genetic component to the disorder. In the present study we used an inbred animal model of ADHD, the spontaneously hypertensive rat (SHR), to investigate the long-term consequences of early life trauma on emotional behaviour in individuals predisposed to developing ADHD-like behaviour.

**Methods:**

We applied a rodent model of early life trauma, maternal separation, to SHR and Wistar-Kyoto rats (WKY), the normotensive control strain from which SHR were originally derived. The effects of maternal separation (removal of pups from dam for 3 h/day during the first 2 weeks of life) on anxiety-like behaviour (elevated-plus maze) and depressive-like behaviour (forced swim test) were assessed in prepubescent rats (postnatal day 28 and 31). Basal levels of plasma corticosterone were measured using radioimmunoassay.

**Results:**

The effect of maternal separation on SHR and WKY differed in a number of behavioural measures. Similar to its reported effect in other rat strains, maternal separation increased the anxiety-like behaviour of WKY (decreased open arm entries) but not SHR. Maternal separation increased the activity of SHR in the novel environment of the elevated plus-maze, while it decreased that of WKY. Overall, SHR showed a more active response in the elevated plus-maze and forced swim test than WKY, regardless of treatment, and were also found to have higher basal plasma corticosterone compared to WKY. Maternal separation increased basal levels of plasma corticosterone in SHR females only, possibly through adaptive mechanisms involved in maintaining their active response in behavioural tests. Basal plasma corticosterone was found to correlate positively with an active response to a novel environment and inescapable stress across all rats.

**Conclusion:**

SHR are resilient to the anxiogenic effects of maternal separation, and develop a non-anxious, active response to a novel environment following chronic mild stress during the early stages of development. Our findings highlight the importance of genetic predisposition in determining the outcome of early life adversity. SHR may provide a model of early life trauma leading to the development of hyperactivity rather than anxiety and depression. Basal levels of corticosterone correlate with the behavioural response to early life trauma, and may therefore provide a useful marker for susceptibility to a certain behavioural temperament.

## Background

There is increasing evidence that environmental factors, particularly stressful events experienced early in life, increase the risk of developing a psychiatric illness and/or a behavioural disorder [1-4]. Following birth, the brain continues to develop, rendering it vulnerable to adverse external influences. These may lead to persistent behavioural, endocrine, and neurochemical changes [5-8]. It is unclear, however, how the physiological and psychological consequences of early life stress are influenced by an inherited predisposition to a developmental disorder, such as attention-deficit/hyperactivity disorder (ADHD).

ADHD is a heterogeneous behavioural disorder characterised by inattention, impulsivity, and hyperactivity [9,10]. It is one of the most common child-psychiatric disorders [11]. Although there is evidence for ADHD having a genetic component, the exact aetiology of the disorder is unknown [10,12]. It is possible that early life experiences interact with the development and expression of an inherited ADHD genotype [10,13].

Maternal separation is a valid model used in research to mimic early life trauma during childhood in humans [7,14-18]. Applied to rats, it involves separating the pups from their mother for a set period of time each day during the first 2 weeks of life. Maternal separation has been found to permanently modify characteristics of the stress response system in offspring, leading to elevated and prolonged stress-induced secretion of corticosterone (the rat equivalent of human cortisol) and adrenocorticotropic hormone [18,19]. These findings of a prolonged endocrine stress response suggest that the stress response system adapts to early life trauma by diminishing negative feedback regulation [18,19]. Most maternal separation paradigms also result in behavioural changes as these rats show increased anxiety-like and/or depressive-like behaviours [16,20,21]. However limited attention has been given to the effects of maternal separation on genetic animal models of human disorders.

While the mechanisms underlying the long-term effects of environmental stress early in life are not known, they are likely to involve activation of intracellular signalling pathways, leading to modifications of the genome, resulting in changes in gene expression and neural function (see [22]). In this way the stressful early-life environment programmes gene expression of the offspring, so that development of a defensive physiological response to stress occurs - a response that would possibly confer protection should the stressful environment persist [22]. Given the influence of early life environmental conditions on gene expression, it is of interest to investigate how the long-term effects of early life trauma are influenced by the individual's genotype. In this view, the long-term effects of maternal separation may differ between different genetic animal models of human disorders. Furthermore, interactions between early life experience and genetic predisposition may account for the variation of the behavioural characteristics of human disorders [23-28].

Another factor influencing the outcome of maternal separation stress may be the sex of the rat. Although many studies have investigated effects of maternal separation in both male and female rats [29-32], there is little consensus on whether one sex is more vulnerable to the effects of postnatal environmental manipulations than the other. It was shown that Sprague-Dawley (SD) females (P28-30) were more resistant to the effects of maternal separation (6 h/day) on measures of impulsive behaviour and cerebral cortical thickness compared to males [31]. Using Wistar rats, an increase in corticotropin-releasing factor-positive cells was found in the paraventricular nucleus of the hypothalamus after an acute swim-stress of maternally separated females, but not males, suggesting that maternal separation increased stress responsivity in females only [29]. Differences in the strain of rat used when investigating the factor of sex on maternal separation effects may account for contradictory findings in the literature.

The present study therefore investigated effects of maternal separation in a rat model of ADHD, the spontaneously hypertensive rat (SHR). The SHR is a well established animal model of ADHD, displaying the three main behavioural characteristics of the disorder: inattention, impulsivity, and hyperactivity, when compared to their normotensive control strain, the Wistar-Kyoto rats (WKY) [33-35], from which they were originally bred [36]. The ADHD-like behaviour of SHR is genetically determined and not a consequence of the SHR dam's behaviour towards the pups, since cross-fostering SHR pups onto WKY or SD dams did not alter their neurochemistry or their behaviour [37]. The use of SHR to investigate how a genetic predisposition to developing ADHD-like behaviour alters the consequences of early life stress is therefore relevant and appropriate. The aim of the present study was to investigate the effects of chronic, mild, developmental stress on behaviour and basal levels of corticosterone in prepubescent male and female rats of different genotypes - the SHR and WKY strains.

## Methods

### Animals

Adult SHR and WKY, originally obtained from Charles River laboratories (USA) and maintained as inbred colonies in the University of Cape Town Animal Unit, were used for breeding of experimental rats. All breeding rats were housed in plexiglass cages in a 12 hour light/dark cycle (lights on from 06h00 to 18h00) with food and water available *ad libitum*, and temperature maintained at 21 - 24°C. All experiments were carried out with approval of the Health Sciences Faculty Research Ethics Committee of the University of Cape Town. Experimental rats used in the present study were SHR (17 males; 19 females) and WKY (18 males, 18 females).

### Experimental design

One to 3 adult females of the same strain were placed in a cage with 1 adult male from the respective strain for 4 days. Following mating, females were housed individually. The date of litter birth was recorded as post-natal day 0 (P0). On P2, the pups were sexed and the litter culled to 8. Alternate litters were assigned to either undergo maternal separation or serve as a non-maternally separated control litter. Maternal separation litters were separated from the dam for 3 hours per day, between 09h00 and 13h00, from P2 to P14. The pups were placed in a clean cage to ensure that the odour of the dam was not present. Non-maternally separated pups, and their dam, were also given a clean cage every day between P2 and P14, to control for handling and a clean environment. At all times handling was kept to a minimum to prevent buffering of the maternal separation effect [38]. Maternal separation pups were housed in a separate room (31 - 33°C to prevent hypothermia) for the 3-hour separation period to prevent mother-infant ultrasonic communication [39], since this may also attenuate maternal separation effects [40]. On P21 all pups were weaned and housed in pairs or threes to prevent isolation stress. On P28 the rats were tested in the elevated-plus maze (EPM). On P30 the rats were placed into the cylinder of water used in the forced swim test (FST) for 15 minutes, and on P31 they were tested in the FST for 5 minutes. The 15-minute habituation phase of the FST is necessary to induce 'learned helplessness' in the rat, so that during the 5-minute test phase, 24 hours later, the rats respond to an inescapable swim rather than anticipate rescue [41]. All behavioural tests were performed in the rat's light phase. On P35, 4 days after the last behavioural test, rats were decapitated between 08h30 and 11h30 and their trunk blood collected for measurement of basal plasma corticosterone levels. Body weights of experimental rats were recorded on P21 and P30.

### Elevated-plus maze

The EPM comprised four arms (45 cm × 10 cm) extending outwards at right angles from a central square area (10 cm × 10 cm), forming a 'plus' shape, elevated 50 cm above the floor. Two of the opposing arms were enclosed on either side by 30 cm high walls (referred to as the closed arms). The other two arms (the open arms) had a 2 cm lip on either side. The ends of all arms were open. The rats were tested in the EPM on P28. In carrying out the test, the rat was placed in the centre of the apparatus facing one of the open arms (the same open arm for the testing of each rat), and left alone in the room to explore the maze for 5 minutes. The maze was cleaned with 10% ethanol between rats to remove olfactory cues. A video camera positioned above the apparatus recorded an aerial view video of each rat. The videos were analysed using Noldus Ethovision, version 5. The parameters recorded by Noldus Ethovision included the total distance travelled (cm), the amount of time (in seconds) spent in the centre area, the closed arms and the open arms, the number of transitions from the centre into the open and/or closed arms, and the maximum velocity achieved by the rat. Each parameter was recorded per minute and for the total 5-minute period. Time spent in the open arms was expressed as a percentage of time spent in the open and closed arms (added together), removing the time spent in the centre. This was to reveal more accurately the level of preference the rat had for the open arms over the closed arms. Open arm entries were similarly expressed as a percentage of entries into the open and/or closed arms to control for the level of activity of the rat. The total arm entries indicate how active the rat was in the apparatus. Thus, expressing the open arm entries as a percentage of total arm entries indicates more accurately the level of preference the rat had for entering the open arms rather than the closed arms.

### Forced swim test

The FST apparatus, a transparent cylinder (diameter = 19 cm, height = 40 cm), contained tap water (25°C, depth = 19 cm). On P30 the pups were placed in the water for a 15 minute habituation period. On P31 (24 hours after the habituation phase) the rats were subjected to a 5-minute swim test in the cylinder. On both days, after removal from the water, the rat was dried under a lamp to prevent hypothermia. The cylinder was rinsed after testing each rat. The 5-minute test phase was recorded using an overhead view camera, since movement of hindlimbs for buoyancy was not needed for analysis [42].

The 5-minute video recordings were analysed according to Cryan et al. [42] and Detke et al. [43]. Behavioural measurements included time spent actively climbing or swimming, and time spent immobile. Every 5 seconds of the video was labelled as swimming (rat making active swimming motions, moving forelimbs in a non-vigorous manner), climbing (rat making vigorous upward directed movements of forelimbs), or immobile (rat making only those movements necessary to keep its head above the water; a 'floating posture'), depending on what the rat spent the majority of the 5 seconds doing. The 5-second intervals of the same behaviour were summed within each minute, to determine the percentage time spent swimming, climbing, and/or immobile for each minute and for the total 5-minute period.

### Corticosterone

On P35 the rats were killed by decapitation between 08h30 and 11h00. Rats of different sexes, strains, and/or treatment groups were decapitated alternately to counterbalance the variability introduced by the long time period during which decapitations took place. During the process rats were housed in a room separate from the laboratory in which decapitations took place. Trunk blood was collected in EDTA-lined tubes and centrifuged at 4°C (4000 rpm, 1537 *g*) for 20 minutes. Plasma aliquots were snap frozen in liquid nitrogen (N_2_) and stored at -80°C. Plasma corticosterone was measured using radioimmunoassay (DEMEDITEC ^125^I Corticosterone RIA from Demeditec Diagnostics GmbH).

### Statistical analysis

All data are expressed as mean ± standard error of the mean (S.E.M.). Data were subjected to Shapiro-Wilk's W test, confirming that all data were normally distributed before applying parametric statistical analysis. All analyses were performed using STASTICA (data analysis software system), version 9.0. Three-way analysis of variance (ANOVA), with treatment (maternal separation), strain and sex as factors, was used to analyse EPM and FST data (the total 5-minute period and each of the 5 minutes), as well as basal levels of corticosterone and weights recorded at P21 and P30. The minute-by-minute analyses were performed to test for differences in behaviour between the groups over the course of the 5 minutes, since minute-by-minute changes in behaviour have been previously reported for the EPM and FST [44-47]. ANOVAs revealing significant effects (p < 0.05) were followed by post-hoc Duncan's tests. Pearson correlation coefficients (r) were calculated for behavioural parameters and basal plasma corticosterone levels, as well as between distance travelled in the EPM and behavioural parameters in the FST.

## Results

Behaviour of the experimental rats was assessed during the 5^th ^week of life (prepuberty) using the elevated plus maze (EPM), a valid test for assessing levels of anxiety-like behaviour [48], and the forced-swim test (FST), a putative assessment of depressive-like behaviour [42], originally derived to screen the efficacy of antidepressant drugs [49,50]. At the end of the 5^th ^week (P35), 4 days after the last behavioural testing day, the rats were killed by decapitation. Their trunk blood was collected in order to measure basal plasma corticosterone levels to determine hypothalamic-pituitary-adrenal (HPA) axis activity in SHR and their control WKY strain. Results show that SHR do not develop increased anxiety-like behaviour following early life trauma as WKY do, but instead respond to the trauma by developing increased behavioural activation, behaviour that correlates positively with high basal plasma corticosterone levels.

### Body weight

Three-way ANOVA of the recorded weights, with treatment (maternal separation), strain and sex as factors, showed a strain effect at P21 (F_(1, 64) _= 10.67, p < 0.01) and at P30 (F_(1, 64) _= 8.58, p < 0.01), but no effect of maternal separation or sex. Duncan's post-hoc test showed that SHR weighed less than WKY at both P21 (p < 0.01) and P30 (p < 0.01).

### Elevated plus maze

#### Time spent in open arms

**Figure 1:**
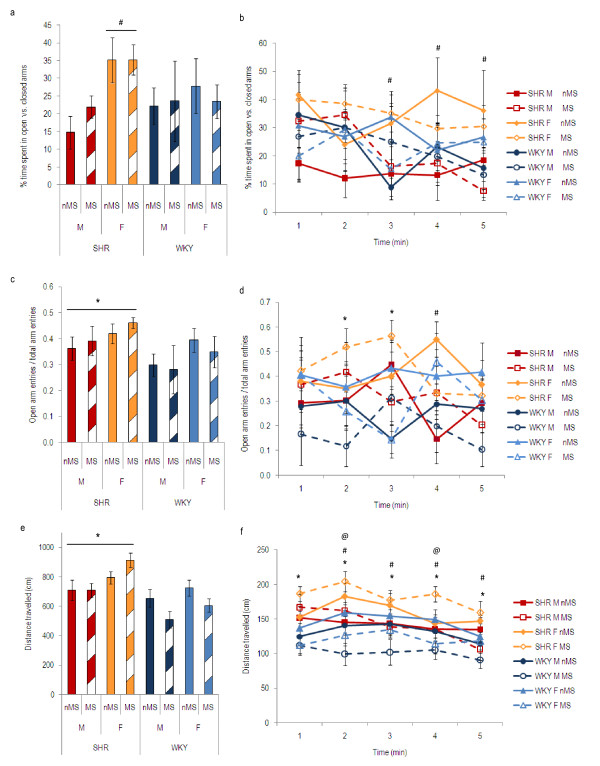
**Elevated plus maze behaviour of SHR and WKY males (M) and females (F), maternally separated (MS) or non-maternally separated (nMS)**. Time spent in the open arms is expressed as a percentage of total time spent in the open and closed arms (a and b). Open arm entries are expressed as a fraction of the total arm entries (c and d). Distance travelled is the total distance travelled in the EPM for the total 5-minute period (e) or within each individual minute (f). For each parameter, data are shown for the total 5-minute period (a, c, and e) and for each individual minute (b, d and f). a. ^#^SHR females spent more of the total 5-minute period in the open arms than SHR males (p < 0.05). b. ^#^Females spent more time in the open arms than males (p < 0.05). c. *SHR entered the open arms more frequently than WKY (p < 0.05). d. *SHR entered the open arms more frequently than WKY (p < 0.05). ^#^Females entered the open arms more frequently than males (p < 0.05). e. *SHR travelled greater distance than WKY (p < 0.05). f. *SHR travelled greater distance than WKY in every minute (p < 0.05). ^#^Females travelled further than males from the 2^nd ^minute onwards (p < 0.01). ^@^Maternal separation decreased distance travelled by WKY (p < 0.05). (SHR males: nMS, *n *= 9, MS, *n *= 9; SHR females: nMS, *n *= 5, MS, *n *= 14; WKY males: nMS, *n *= 9; MS, *n *= 9; WKY females, nMS, *n *= 10, MS, *n *= 8).

Three-way ANOVA (treatment, strain and sex as factors) of the time spent in the open arms of the EPM, expressed as a percentage of total time spent in open and closed arms, revealed a significant sex effect (F_(1, 64) _> 4.30, p < 0.05) in the total 5-minute period, and the in 3^rd^, 4^th ^and 5^th ^minutes of the test, but no effect of maternal separation. Post-hoc analysis of the total 5-minute period revealed that females spent more time in the open arms than males (p < 0.05) which was largely due to the SHR females spending more time in the open arms than SHR males (p < 0.05, figure 1a). Post-hoc analysis of individual minutes showed that females spent more time in the open arms than males in the 3^rd^, 4^th ^and 5^th ^minutes of the 5-minute test (p < 0.05, figure 1b).

#### Open arm entries

Three-way ANOVA (treatment, strain and sex as factors) of entries into the open arms of the EPM, expressed as a percentage of total EPM arm entries, showed a strain effect (F_(1, 64) _> 3.99, p < 0.05) and a sex effect (F_(1, 64) _> 4.09, p < 0.05) in the total 5-minute period. Analysis of the individual minutes showed the strain effect to be present in the 2^nd ^and 3^rd ^minutes, and the sex effect in the 4^th ^minute. The analysis also showed a strain*treatment interaction (F_(1, 64) _= 4.17, p < 0.05) in the 2^nd ^minute and a strain*sex*treatment interaction (F_(1, 64) _= 7.91, p < 0.01) in the 3^rd ^minute suggesting that the effects of maternal separation on open arm entries differed in SHR and WKY. Post-hoc analysis of the total 5-minute period revealed that SHR entered the open arms more frequently than WKY (p < 0.05, figure 1c) and that females entered the open arms more frequently than males. Post-hoc analysis of individual minutes showed SHR entered the open arms more frequently than WKY in the 2^nd ^and 3^rd ^minutes (p < 0.05, figure 1d). Females entered the open arms more frequently than males in the 4^th ^minute of the test (p < 0.05, figure 1d).

### Distance travelled

Three-way ANOVA (treatment, strain and sex as factors) of the distance travelled in the EPM revealed a significant strain effect (F_(1, 64) _> 4.58, p < 0.05), a sex effect (F_(1, 64) _> 4.13, p < 0.05), and strain*treatment interaction (F_(1, 64) _> 4.95, p < 0.05) in the total 5-minute period of the test. Analysis of the individual minutes showed that the strain effect was present for each of the 5 minutes of the test, the sex effect was present from the 2^nd ^minute onwards, while the strain*treatment interaction was present in the 1^st^, 2^nd ^and 4^th ^minutes of the test. Post-hoc analysis of the total 5-minute period revealed that SHR travelled further than WKY (p < 0.05, figure 1e), females covered a greater distance than males (p < 0.01), and maternal separation decreased the distance travelled by WKY, but not SHR (p < 0.05). Post-hoc analysis of the individual minutes showed that in each minute SHR travelled further than WKY (p < 0.05, figure 1f), females travelled further than males from the 2^nd ^minute onwards (p < 0.01, figure 1f), and maternally separated WKY travelled significantly less distance than non-maternally separated WKY in the 2^nd ^and 4^th ^minutes of the test (p < 0.05, figure 1f).

### Forced-swim test

#### Time spent immobile

**Figure 2:**
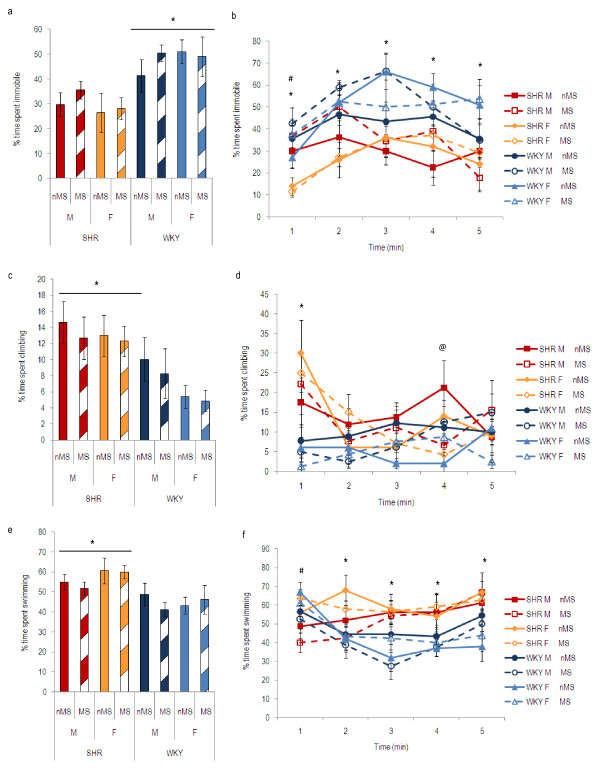
**Forced swim test behaviour of SHR and WKY males (M) and females (F), maternally separated (MS) or non-maternally separated (nMS)**. Time spent immobile (a and b), climbing (c and d) or swimming (e and f) is expressed as a percentage of total time. For each parameter, data are shown for the total 5-minute period (a, c, and e) and for each individual minute (b, d and f). a. *WKY spent more of the total 5-minute period immobile than SHR (p < 0.01). Maternal separation did not increase time spent immobile in either strain. b. *WKY spent more time immobile than SHR (p < 0.01). ^#^SHR males spent more time immobile than SHR females in the 1^st ^minute (p < 0.001). c. *SHR spent more of the total 5-minute period climbing than WKY (p < 0.05). d. *SHR spent more time climbing than WKY in the 1^st ^minute (p < 0.001). ^@^Maternal separation increased climbing in SHR in the 4th minute (p < 0.01). e. *SHR spent more of the total 5-minute period swimming than WKY (p < 0.05). f. *SHR spent more time swimming than WKY (p < 0.05). ^#^Females spent more time swimming during the 1^st ^minute than males (p < 0.01). (SHR males: nMS, *n *= 9, MS, *n *= 9; SHR females: nMS, *n *= 5, MS, *n *= 14; WKY males: nMS, *n *= 9; MS, *n *= 9; WKY females, nMS, *n *= 10, MS, *n *= 8).

Three-way ANOVA (treatment, strain and sex as factors) of time spent immobile in the FST, expressed as a percentage of total time in the FST, showed a strain effect (F_(1, 63) _> 9.18, p < 0.01) in the overall 5-minute period. Analysis of the individual minutes showed the strain effect to be present in each of the 5 minutes, and a sex effect (F_(1, 63) _= 10.83, p < 0.01) present in the 1^st ^minute of the test. Post-hoc analysis of the total 5-minute period revealed that WKY spent more time immobile than SHR (p < 0.01, figure 2a). Post-hoc analysis of the individual minutes showed WKY were more immobile than SHR in each of the 5 minutes (p < 0.01, figure 2b). Furthermore, SHR males were more immobile than SHR females during the 1^st ^minute (p < 0.001, figure 2b). Maternal separation did not increase immobility in either strain (figure 2b).

#### Time spent climbing

Three-way ANOVA (treatment, strain and sex as factors) of time spent climbing in the FST, expressed as a percentage of total time in the FST, showed a strain effect (F_(1, 63) _> 12.41, p < 0.001) in the total 5-minute period. Analysis of the individual minutes showed the strain effect to be present in the 1^st ^minute of the test only. A strain*treatment interaction (F_(1, 63) _= 6.55, p < 0.05) was present in the 4^th ^minute of the test, suggesting that maternal separation affected the climbing behaviour of SHR and WKY in different ways. Post-hoc analysis of the total 5-minute period showed that SHR spent more time climbing than WKY (p < 0.01, figure 2c). Post-hoc analysis of the individual minutes showed that SHR spent more of the 1^st ^minute climbing than WKY (p < 0.001, figure 2d) and maternal separation decreased SHR climbing during the 4^th ^minute of the test (p < 0.01, figure 2d).

#### Time spent swimming

Three-way ANOVA (treatment, strain and sex as factors) of time spent swimming in the FST, expressed as a percentage of total time in the FST, revealed a strain effect (F_(1, 63) _> 7.51, p < 0.01) in the total 5-minute period. Analysis of the individual minutes showed the strain effect was present from the 2^nd ^minute onwards. The analysis also revealed a sex effect (F_(1, 63) _= 8.55, p < 0.01) present in the 1^st ^minute only. Post-hoc analysis of the total 5-minute period showed that SHR spent more time swimming than WKY (p < 0.05, figure 2e). Post-hoc analysis of the individual minutes showed, from the 2^nd ^minute onwards, SHR spent more time swimming than WKY (p < 0.05, figure 2f) and females spent a greater percentage of the 1^st ^minute swimming than males (p < 0.01, figure 2f).

### Correlation between locomotor activity in the EPM and behaviour in the FST

Pearson correlation coefficients (r) were calculated for distance travelled in the EPM and behaviour in the FST. Distance travelled in the EPM correlated i) negatively with time spent immobile in the FST (r = -0.45, p < 0.001, Table 1), ii) positively with time spent climbing in the FST (r = 0.31, p < 0.05, Table 1), and iii) positively with time spent swimming in the FST (r = 0.40, p < 0.001, Table 1).

**Table 1:** Pearson correlation coefficients between distance travelled in the EPM and behaviour in the FST

Distance travelled						
	**Forced-swim test**					

	**Time spent immobile**		**Time spent climbing**		**Time spent swimming**	
	**r(x,y)**	**p**	**r(x,y)**	**p**	**r(x,y)**	**p**

1st minute	-0.36*	**0.003**	0.43*	**0.0002**	-0.04	0.723
2nd minute	-0.49*	**0.00002**	0.29*	**0.020**	0.41*	**0.006**
3rd minute	-0.21	0.098	0.06	0.652	0.19	0.121
4th minute	-0.30*	**0.013**	-0.06	0.635	0.37*	**0.002**
5th minute	-0.12	0.328	0.04	0.754	0.15	0.23
Total time	-0.45*	**0.0001**	0.31*	**0.012**	0.40*	**0.0007**

*Distance travelled in the EPM correlated positively with time spent climbing and swimming in the FST, while it correlated negatively with time spent immobile in the FST (p < 0.05).

### Plasma corticosterone

**Figure 3:**
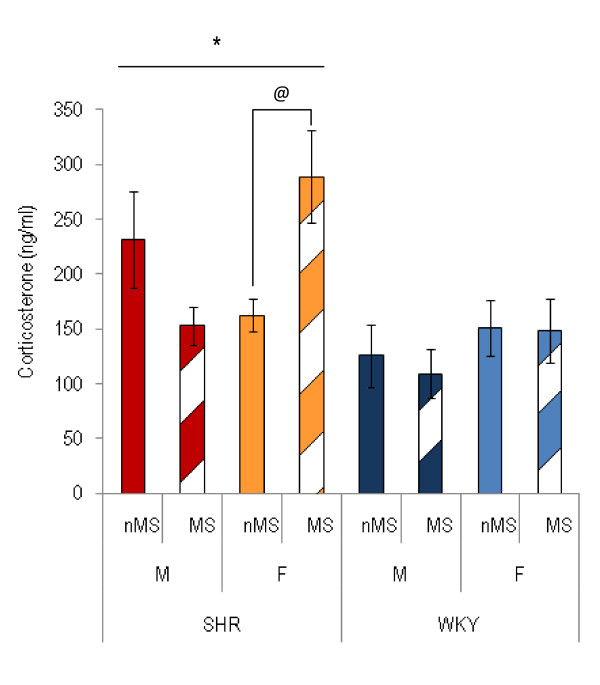
**Basal plasma corticosterone levels of SHR and WKY males (M) and females (F), maternally separated (MS) or non-maternally separated (nMS)**. *SHR had higher basal levels of corticosterone than WKY (p < 0.001). ^@^Maternal separation increased basal levels of corticosterone in SHR females (p < 0.05). (SHR males: nMS, *n *= 5, MS, *n *= 9; SHR females: nMS, *n *= 4, MS, *n *= 14; WKY males: nMS, *n *= 7; MS, *n *= 9; WKY females, nMS, *n *= 10, MS, *n *= 6).

Three-way ANOVA (treatment, strain and sex as factors) of the basal plasma corticosterone levels of SHR and WKY showed a significant strain effect (F_(1, 57) _= 8.36, p < 0.01), and a sex*treatment effect (F_(1, 57) _= 4.38, p < 0.05). Post-hoc analysis showed that basal levels of corticosterone of SHR were higher than that of WKY (p < 0.001, figure 3), and maternal separation increased basal levels of corticosterone in SHR females (p < 0.05, figure 3).

### Correlations between behaviour and plasma levels of corticosterone

Pearson correlation coefficients (r) were calculated for basal plasma corticosterone levels and behaviour in the EPM and FST. Basal plasma corticosterone levels correlated i) positively with time spent in the open arms of the EPM during the 5^th ^minute (r = 0.30, p < 0.05, Table 2), ii) positively with distance travelled in the EPM (r = 0.39, p < 0.01, Table 2), iii) negatively with time spent immobile in the FST (r = -0.30, p < 0.05, Table 2), iv) positively with time spent climbing in 1^st ^minute of the FST (r = 0.30, p < 0.05, Table 2), and v) positively with time spent swimming in the FST (r = 0.37, p < 0.01, Table 2).

**Table 2:** Pearson correlation coefficients between basal plasma corticosterone and behaviour in the EPM and the FST

Basal plasma corticosterone						
	**Elevated plus maze**					
	
	**Time in open arms**		**Open arm entries**		**Distance travelled**	
	
	**r(x,y)**	**p**	**r(x,y)**	**p**	**r(x,y)**	**P**

1st minute	0.11	0.377	0.14	0.246	0.29*	**0.016**
2nd minute	-0.06	0.634	0.01	0.935	0.42*	**0.0003**
3rd minute	0.14	0.250	0.22	0.076	0.27*	**0.027**
4th minute	0.2	0.112	0.05	0.693	0.35*	**0.003**
5th minute	0.30*	**0.013**	0.07	0.600	0.27*	**0.026**
Total time	0.2	0.112	0.14	0.269	0.39*	**0.001**

	**Forced-swim test**					
	
	**Time spent immobile**		**Time spent climbing**		**Time spent swimming**	
	
	**r(x,y)**	**p**	**r(x,y)**	**p**	**r(x,y)**	**P**

1st minute	-0.27*	**0.030**	0.30*	**0.014**	-0.02	0.891
2nd minute	-0.36*	**0.032**	0.11	0.369	0.35*	**0.004**
3rd minute	-0.42*	**0.0004**	0.01	0.937	0.28*	**0.022**
4th minute	-0.11	0.360	0.16	0.206	0.39*	**0.001**
5th minute	-0.38*	**0.002**	-0.12	0.317	0.21	0.090
Total time	-0.30*	**0.014**	0.19	0.123	0.37*	**0.002**

*Basal plasma corticosterone correlated positively with time spent in the open arms in the 5^th ^minute of the EPM, distance travelled in the EPM, time spent climbing in the 1st minute of the FST, and time spent swimming in the FST, while it correlated negatively with time spent immobile in the FST (p < 0.05).

## Discussion

Results from the current study show that maternal separation affects anxiety-like behaviour and locomotor activity of SHR and WKY in different and possibly opposing ways. Maternal separation increased the distance travelled in the EPM and number of entries into the open arms made by SHR, particularly in the females, while it had the opposite effect on WKY, suggesting that SHR developed behaviour that was more active and devoid of anxiety following early life trauma, while WKY developed increased anxiety-like behaviour that was less active following early life trauma. The increased anxiety-like behaviour and decreased behavioural activity observed in WKY following maternal separation agrees with reported effects of maternal separation on other rat strains [16,51].

The distance travelled in the EPM reflects activity within a novel environment, and therefore cannot be used as a measure of activity on its own, due to the confounding effect of anxiety. Therefore, changes in distance travelled in the EPM due to maternal separation may be due to altered levels of anxiety and/or altered levels of activity per se. In SHR, maternal separation appeared to increase activity in the EPM, decreasing anxiety-like behaviour. This effect was most notable in the female SHR. This is an important finding considering evidence in the literature showing that the long-term effects of childhood trauma are variable, and highly dependent on the individual [13,24-26,52,53]. Studies have shown that early life trauma is associated with increased risk of developing a number of different disorders later in life, including anxiety disorders and/or depression, conduct disorder, hyperactivity, psychotic symptoms and ADHD [2,13,24-26,52-57]. The disorders which arise in response to environmental adversity during development are thus highly dependent on an individual's genotype [23-27]. Furthermore, it has been shown that genetic predisposition may confer resilience to the development of a disorder following childhood trauma [24,26]. A study by Polanczyk et al. [25] found that a haplotype in the corticotrophin releasing hormone receptor gene 1 was associated with protective effects against adult depression in individuals who reported maltreatment during childhood (assessed by the Childhood Trauma Questionnaire). Caspi et al. [26] showed that children with a functional polymorphism in the promoter region of the monoamine oxidase A (MAOA) gene, leading to high levels of MAOA expression, were less likely to develop antisocial behaviour following childhood maltreatment. It has also been shown, by Stevens et al. [24], that the long term effects of early life deprivation (reared in deprived conditions from infancy for more than 6 months before being adopted) on ADHD-like behaviour in childhood to mid-adolescence is moderated by polymorphisms of the dopamine transporter (DAT1) gene.

Our findings contribute to the literature highlighting the importance of genetic predisposition on the outcome of early life adversity. SHR may provide a genetic animal model for the instances when early life trauma leads to the development of abnormal behaviours that are not marked by anxiety and/or depression, but rather by increased activity in a novel environment and decreased anxiety.

Although maternal separation altered the number of open arm entries in the EPM made by SHR and WKY, suggesting altered anxiety-like behaviour, there was no effect of maternal separation on time spent in the open arms of the EPM, a second measure of anxiety-like behaviour. Maternal separation also had no effect on depressive-like behaviour, measured by time spent immobile in the FST, in SHR or WKY. This is contrary to studies showing decreased time spent in open arms [16,51] and diminished activity in the FST [17,58] in other rat strains due to maternal separation. There have, however, also been studies in the literature reporting no effect of maternal separation on open arm entries and time spent in the open arms of the EPM [32,58]. The mentioned studies all focused on the behavioural consequences of maternal separation in adulthood, and did not use SHR or WKY. It is possible that anxiety-like and depressive-like behavioural characteristics that result from maternal separation manifest fully in adulthood, and are only partially expressed in prepubescent rats, or that maternal separation effects are highly strain-dependent.

Consistent with their hyperactive phenotype, SHR travelled a greater distance in the EPM and spent less time immobile in the FST than WKY, as reported in previous studies [37,59,60]. Since the environment of the EPM was novel to the animals in the present study, distance travelled in the EPM provided a measure of exploratory behaviour [61]. Decreased distance travelled by WKY due to maternal separation implies increased passive, inhibited behaviour. WKY typically show high levels of behavioural inhibition [62,63], characterised by a reserved response or inactivity in the face of novelty [64]. In the literature they are reported to show decreased ambulation and high behavioural passivity in a novel environment compared to other strains [65,66]. SHR, on the other hand, show high behavioural activation, characterised by approach motivation and spontaneity [67], and respond actively in a novel situation [37]. Maternal separation tended to increase the active response of SHR to a novel environment, while it accentuated the inhibited behaviour of WKY, thereby amplifying the genetically predisposed behavioural temperaments of the strains. This finding suggests that the early life experience of separation from the dam influenced development of the behavioural inhibition and/or behavioural activation systems.

Studies have shown that children exposed to an unstable and/or traumatic environment very early in life are at a greater risk of developing chronic behavioural inhibition in childhood [68,69]. Contrary to this, however, it has also been shown that children exposed to early life stress are at a higher risk of developing behavioural disinhibition [70]. The long term effects of early life trauma on the behavioural inhibition/disinhibition profile of a child may be dependent on the child's genetic predisposition [68,70]. In one study it was shown that children are more likely to develop behavioural inhibition during middle childhood if they are raised in a low social support environment, and have a specific polymorphism in the promoter region of the gene for the serotonin transporter [68]. Another study found that the MAOA functional promoter polymorphism interacts with family adversity and stressful events experienced early in life to influence behavioural disinhibition in children, marked by hyperactivity and conduct disturbances [70]. In the present study the rat strain predisposed to developing ADHD-like behaviour responded to early life trauma with an increase in disinhibited/active behaviour, while their control strain developed increased behavioural inhibition. These results suggest that genetic predisposition interacts with the environment giving rise to either increased behavioural inhibition or increased behavioural disinhibition/activation.

It has been hypothesised that behaviour in the FST reveals the animal's coping strategy when confronted with an inescapable stressor [65,71]. While SHR adopted a more active strategy (higher climbing and swimming behaviour), displaying behavioural activation, WKY responded in a more passive manner, showing typical behavioural inhibition. The most dramatic difference in FST behaviour between SHR and WKY was apparent in the first minute, when SHR spent a greater percentage of the time climbing than WKY. The strain effect in climbing behaviour apparent for the total 5-minute period was due solely to the significant difference between the strains in the first minute. Climbing behaviour did not differ significantly between the two strains from the second minute onwards. This finding demonstrates that the initial response to an acute, inescapable stress may be determined by genetic predisposition.

It has been observed that, while having similar effects on time spent immobile in the FST, different antidepressant drugs produce characteristic differential effects on the active behaviours in the FST, depending on the neurotransmitter systems which they target [41,49,72]. While selective serotonin reuptake inhibitors (SSRIs), such as fluoxetine, sertaline, and paroxetine, increase levels of swimming in the FST, drugs that block the reuptake of norepinephrine, such as desipramine, and maprotiline, increase climbing behaviour in the FST [41,49,71,73]. Supporting these findings is a study by Brenes et al. [74], in which hippocampal concentrations of norepinephrine and serotonin were measured in SDs using high-performance liquid chromatography coupled with electrochemical detection, and then correlated with behaviour in the FST. They found that hippocampal norepinephrine concentrations correlated positively with climbing behaviour and negatively with immobility, while hippocampal serotonin concentration correlated positively with swimming behaviour and negatively with immobility [74].

Attaching neurochemical profiles to active behaviours in the FST has allowed researchers to infer which neurotransmitter systems are involved in the antidepressant-like effects of novel treatments [41]. Although time spent immobile differed between SHR and WKY in each minute of the 5-minute FST, the difference in the first minute was due to SHR spending significantly more time climbing. From the second minute onwards, differences in immobility between the strains were due to SHR spending more time swimming. It is possible that in response to the acute stress, SHR had a greater and more immediate release of norepinephrine into various parts of the brain, possibly due to decreased autoreceptor-mediated inhibition of norepinephrine release [75], resulting in increased climbing behaviour relative to WKY. This agrees with the physiological role of norepinephrine and its release in response to stress [76,77], and with *in vitro *superfusion experiments showing that release of norepinephrine in the prefrontal cortex and hippocampus in response to a pulse of glutamate is significantly greater in SHR compared to WKY [37,75,78,79]. Strain differences in behaviour from the second minute onwards could reflect lower basal levels of serotonin in WKY brain relative to SHR [41]. These suggestions are, however, speculative, and require further investigation.

The climbing behaviour of non-maternally separated SHR increased dramatically from the 3^rd ^to the 4^th ^minute of the FST, while that of maternally separated SHR did not, resulting in a significant treatment effect in SHR during this minute. The sudden increase in climbing of non-maternally separated SHR could reflect a second attempt to escape, an attempt that was absent in those SHR that had experienced mild, chronic, developmental stress. This result could indicate a change in the coping strategy of SHR due to the maternal separation stress. However, further research is required. Maternal separation had the opposite effect on WKY climbing behaviour in the 4^th ^minute, tending to increase climbing behaviour in this minute. While the neurochemical basis of this effect of maternal separation is not known, the result indicates opposing effects of chronic, mild, developmental stress on the same behaviour of the two different rat strains.

The high levels of immobility, also referred to as learned helplessness, exhibited by WKY in the FST, is in agreement with the literature [59,60,71], and with their passive behaviour in the EPM. Based on results such as these, WKY has been proposed as an animal model for depression [65,80]. Other studies suggest that the passive response of WKY in behavioural assays indicates behavioural inhibition, and propose WKY as an animal model of anxiety vulnerability rather than depression [62,63]. Since maternal separation had no effect on immobility time of WKY in the FST, but did enhance WKY's passivity in the EPM, it appears that maternal separation increased behavioural inhibition of WKY in the face of a novel environment (EPM), but not acute stress (FST).

Antidepressant drugs that block re-uptake of norepinephrine in the brain, but not those that block the re-uptake of serotonin, are effective in decreasing immobility in the FST in WKY [71,81] and increasing locomotor activity of WKY in the open field test [81], thereby increasing their behaviour towards that of SHR. Since SHR were originally derived from WKY [36], the two strains may still maintain some similarities in their neurochemistry. The differences in the neurochemistry between the two strains that have resulted in SHR being more active and displaying symptoms of ADHD may be related to the noradrenergic system [37,75,78,79]. Furthermore, the effect of maternal separation on levels of behavioural inhibition and activation in SHR and WKY may be related to alterations in noradrenergic transmission.

Sex effects in the EPM showed that females were more active and less anxious than males, in agreement with previous studies [32,37,82,83]. Females spent more time swimming than males in the FST, and the SHR females spent less time immobile than SHR males. These results indicate that females respond in a more active way to an acute, inescapable stress.

High locomotor activity in the novel environment of the EPM correlated positively with active behaviour in the FST across both strains, and negatively with immobility in the FST. The positive correlation suggests that the ability to respond actively in the two different behavioural assays may depend on similar brain circuitry. Furthermore, basal plasma corticosterone correlated positively with distance travelled in the EPM, swimming in the FST, and climbing in the first minute of the FST, and correlated negatively with immobility in the FST. This suggests that basal plasma corticosterone correlates positively with an active response to a novel environment and to an inescapable stress, and correlates negatively with a passive, ambivalent response.

While SHR were found to have elevated basal plasma corticosterone compared to WKY, this finding was likely due to the increased basal plasma corticosterone of SHR females (see figure 3). Maternal separation increased basal levels of plasma corticosterone in SHR females only, demonstrating that effects of chronic mild developmental stress are not only strain-dependent, but also sex-dependent. In a study using Wistar rats, Desbonnet et al. [29] found an increase in CRF cells in the PVN due to maternal separation, in females only. Renard et al. [30] found, also using Wistar rats, reduced HPA axis response to an acute stress in males following maternal separation. While not significant, the HPA axis response to an acute stress appeared heightened due to maternal separation in females [30]. The present study, therefore, contributes to the evidence in the literature for sexually divergent effects of maternal separation on regulation of plasma stress hormones. Since the active response by SHR females in both the EPM and FST in the present study was not influenced negatively by maternal separation, but tended to increase, our results suggest that SHR females adapted to the early life trauma by increasing their behavioural activation, maintaining an active response to the stress of a novel situation or inescapable swim stress. Developmental changes in the locus-coeruleus-norepinephrine system and the HPA axis may have occurred, which is reflected in their persistently high behavioural activation [77]. This adaptive mechanism may have required, or given rise to, the increase in basal corticosterone levels present in maternally separated SHR females.

Since SHR did weigh significantly less than WKY at the time of testing, it is arguable that the strain differences in behaviour are related to differences in weight rather than strain. It is also possible that the difference in weight between strains was due to differences in behaviour - i.e. SHR weighed less than WKY because they were far more active. The strain effects evident in the results do, however, agree with the literature. Since maternal separation and sex had no effect on weight, treatment effects and sex effects present in the results cannot be attributed to differences in weight.

## Conclusion

In conclusion, our results suggest that SHR and WKY may be appropriate genetic models for the opposing effects of early life trauma on the behavioural inhibition and behavioural activation systems. It also appears that basal levels of corticosterone are associated with the variation in behavioural response to early life trauma, and may be useful as a marker for susceptibility to a certain behavioural temperament. Further research is required, however, to establish the effects of maternal separation on behavioural inhibition versus behavioural disinhibition in these strains, and to establish the role of basal plasma corticosterone. Investigating these strains with this novel hypothesis in mind may allow establishment of risk factors for the development of various disorders characterised by increased behavioural inhibition or disinhibition that arise following traumatic experiences early in life.

## Abbreviations

ADHD: attention-deficit/hyperactivity disorder; SHR: spontaneously hypertensive rat; WKY: Wistar-Kyoto rats; MS: maternally separated; nMS: non-maternally separated; EPM: elevated-plus maze; FST: forced swim test; ANOVA: analysis of variance; HPA: hypothalamic-pituitary-adrenal; MAOA: monoamine oxidase A;

## Competing interests

The authors declare that they have no competing interests.

## Authors' contributions

TS contributed to the design of the study, carried out the experimental procedures, analysed the data, and drafted the manuscript. FMH and VAR developed and designed the study, supervised the execution of the study, and assisted with drafting the manuscript. All authors have read and approved the final manuscript.
